# Racial and Ethnic Disparity in the Administration of General Anesthesia

**DOI:** 10.26502/acc.073

**Published:** 2024-10-28

**Authors:** Fihr Chaudhary, Devendra K Agrawal

**Affiliations:** Department of Translational Research, College of Osteopathic Medicine of the Pacific,Western University of Health Sciences, Pomona CA 91766, USA

**Keywords:** Cesarean Delivery, Ethnic Diversity, General Anesthesia, Healthcare Inequities, Implicit Bias, Maternal Outcomes, Minority Health Disparities, Neuraxial Analgesia, Obstetric Anesthesia, Pediatric Anesthesia, Perioperative Care, Preoperative Care, Racial Disparities, Socioeconomic Factors, Surgical Outcomes

## Abstract

Healthcare disparities continue to affect communities in the United States that are racially and ethnically diverse, disabled, and economically disadvantaged, even though medical and technological advancements have made great strides in these areas. Disparities in health outcomes and difficulties obtaining care for both acute and chronic illnesses are more common among these populations when compared to the overall population. Disparities in anesthesia care delivery have been documented in multiple studies, and they are based on factors such as patients’ racial/ethnic background, gender, sexual orientation, ability to communicate in English, and accessibility to health insurance. Despite this, there are limited reports in academic journals on the differences in general anesthesia. This article provides a critical review of literature on racial and ethnic disparities in the use of general anesthesia for adults having obstetric, general, or pediatric surgeries, as well as for their preoperative, intraoperative, and postoperative anesthesia care.

## 1. Introduction

Following the publication of the Heckler report in the 1980s by the Department of Health and Human Services, minority health gained significant national attention [1]. This study examined the magnitude and relevance of minority health disparities in the United States for the first time. About sixty thousand additional deaths occur each year in the United States because of health disparities, according to this research by the Task Force on Black and Minority Health [1]. Research on the health of minority populations exploded in the last 40 years and indeed minorities experience worse health outcomes. Over 74,000 Black Americans died prematurely between 2016 and 2018 [2], despite increased focus on these disparities. Black Americans continue to have a higher all-cause mortality rate than White Americans.

In current clinical practice, general anesthesia may be administered for a variety of reasons, such as when neuraxial anesthesia is insufficient, when time is of the essence and neuraxial anesthesia cannot be properly administered, or when a patient expressly requests or requires a different type of anesthesia. For better maternal morbidity and outcomes, one practical clinical intervention could be to decrease the use of general anesthesia when it may not be necessary [3]. There are a lot of risks connected with general anesthesia, including infections at the surgical site, venous thromboembolic events, and anesthetic problems (up to 44%) [3].

## 2. Disparities in the Receipt of General Anesthesia for Cesarean Delivery

The rates of racial differences in the use of general anesthesia for caesarean deliveries and labor analgesia stand out [4]. For caesarean deliveries, non-Hispanic Black (henceforth Black) and Hispanic patients are more likely to have general anesthesia than non-Hispanic White (henceforth White) patients [5]. There are limited reports on the causal factors for this discrepancy. Unfortunately, most of the studies used population-level analyses lacking detail while investigating the racial and ethnic differences in the use of anesthesia during caesarean sections.

There may be racial and cultural differences in the reasons for general anesthesia during caesarean birth and risk factors for such a procedure, but this 00,,remains an open question [3,4,5]. To enhance mother outcomes and reduce disparity gaps, it is important to identify and treat the cause of racial and ethnic anesthetic inequalities. Here we will look at racial and ethnic disparities in the indications for and rates of general anesthesia for caesarean deliveries.

It has been found that there are racial and ethnic differences in the use of general anesthesia for caesarean deliveries [5]. This confirms previous research that found Black patients to be nearly twice as likely as White patients to have a caesarean section while under general anesthesia [5,6]. The results were further supported by showing that the use of labor epidural analgesia before intrapartum caesarean delivery does not affect the outcome based on race or ethnicity. Since neuraxial catheters enable for the conversion of labor analgesia to surgical anesthesia for caesarean birth, the discovery that racial and ethnic differences are reduced among patients with these catheters in situ during labor is both novel and clinically significant. Timely conversion of neuraxial labor analgesia to anesthesia is one technique for avoiding an avoidable general anesthetic if an urgent or intrapartum caesarean birth is necessary [7].

There is racial disparity in the administration of general and neuraxial labor analgesics, with Black and Hispanic patients receiving lower rates of administration compared to White individuals [8]. Black people are disproportionately uninsured and have lower rates of private insurance compared to White people [9]. Having health insurance improves access to care, outcomes for specific conditions, and self-reported health significantly [10]. Systemic variables, social determinants of health, clinical factors (implicit and explicit prejudice, for example), and communication hurdles are likely all contributing to the unequal rates of neuraxial analgesic use [11].

Although there is no definitive answer regarding the ideal rate of general anesthesia for caesarean deliveries, national organisations like the Society for Obstetric Anaesthesia and Perinatology have set standards and guidelines, with the aim of achieving a rate of less than 5% [12]. One strategy to reduce anesthesia-related inequalities could be to gather patients’ preferences for neuraxial analgesia and inform them of their alternatives for labor analgesics early on [13]. Furthermore, following finding that general anesthesia rates were equal across racial and ethnic groups when an epidural was in place for an intrapartum caesarean birth, researchers may want to compare the two types of caesarean sections for obstetrical reasons in future research. Other potentially actionable characteristics related to non-intrapartum caesarean deliveries should also be investigated through the prism of health care equity.

## 3. Racial Inequity in Pediatric Anesthesia

In the United States, numerous racially charged incidents in 2020 brought the issue of minority health disparities back into the spotlight. While most of the attention has been on adults, these disparities also affect children. It is our hope that this analysis of racial inequities in pediatric perioperative treatment would inspire the anesthesia community to join the struggle against structural racism.

Regrettably, these discrepancies can affect children as well. One example of persistent disparities in infancy is infant mortality. While infant mortality has decreased in the United States over the last several decades, the disparity between Black and White children has widened; the incidence of death for Black infants is double that of White infants. Additionally, compared to White newborns, the infant mortality rate is greater for racial and ethnic minority infants, such as Native American and Native Hawaiian infants [14]. While there is a lack of research on pediatric health disparities compared to adult populations, there are clear disparities in the way juvenile comorbidities and mental health are diagnosed, treated, and the results they accomplish [14].

Because of our limited contact with patients and the perioperative context, the role of anesthesiologists in eradicating healthcare inequities is inadequately defined. While there have been reports of anesthetic delivery differences [15], many minority health studies have shown that minorities face difficulties in gaining access to care and have worse surgical outcomes [14, 16]. Nevertheless, anesthesiologists must be cognizant of perioperative disparities as well as individual and systemic factors influencing minority children’s health for them to deliver the best care possible. Perioperative care providers, including anaesthesiologists, have a responsibility to their patients to address systemic racism and its effects on minority health because the healthcare industry reflects society at large. We need to focus on systemic factors rather than individual ones when trying to solve health disparities. In this section, we will examine the racial disparities in children’s perioperative health and management, talk about the factors that lead to these inequalities, and evaluate the literature on the topic.

### 3.1 Racial Disparities in Preoperative Care

There is a lack of research that focusses specifically on anesthesia concerning disparities. But to think that there are no racial or ethnic differences in the way anaesthesia is administered is misleading. One article in 2019 showed that Black youngsters were “adultified” in the preoperative setting [17]. The social principle of adultification states that racial factors influence how we conceptualize childhood; for example, White people tend to view Black children as more developed and less dependent on their carers than their Black counterparts [17, 18]. The study indicated that compared to White children, Black children <5 years old were less likely to receive oral anxiolytics and Black children <15 years old were less likely to have a parent present during the induction of anesthesia. Worryingly, these results indicate that the Black youngster is becoming “adultified” in the preoperative setting. Anesthesiologists are human and prone to biases, both unconscious and conscious, when it comes to administering pre-medication and selecting anesthesia induction techniques. It is possible that anesthesiologists will overlook Black children’s preoperative anxiety because they perceive them as more mature than White children. Also, compared to White children, Black children are more likely to have adverse perioperative outcomes, and this could be due to the difference in induction approach. In the APRICOT research, a European prospective multicenter investigation, inhalational induction was linked to an increased risk of serious critical respiratory episodes when contrasted with intravenous induction [19]. Anesthesiologists can take concrete steps to reduce the racial gap in postoperative outcomes by using measures such as premedication with midazolam or changing the manner of induction of anesthesia (mask versus intravenous).

Contrary to one study that looked at pre-medication and induction techniques, another paper examined the amounts of medication given to Latino and White children aged 18 and younger who had tonsillectomy and adenoidectomy performed at one hospital between 2003 and 2005 [20]. It was shown that White children were more likely to be premedicated than Latino children; nevertheless, Latino children were more likely to have a parent present during anaesthesia induction. When comparing Latino and White youngsters, there was no discernible difference in the induction route (inhalational vs. intravenous). Separately, one paper looked at variations in the amount of anesthetic given to children having appendectomy at one facility from 2010 to 2015 [21]. When comparing White and Black children, the likelihood of receiving midazolam prior to medicine was higher for the former. However, no significant difference in premedication by race was found after adjusting for patient age, gender, and heterogeneity in anesthesiologist prescription practices.

The studies do not take into consideration whether the premedication and parental presence were provided without bias based on race or ethnicity. It is possible that cultural variations in care preferences or comprehension of the intervention explain why some patients use these preoperative measures more than others, even when these interventions are made available to all patients equally.

## 4. Intraoperative Racial Disparities

### 4.1 Anesthetic Administration

Racial inequality in perioperative care has been the subject of relatively few investigations. No statistically significant difference was identified between the administration of lidocaine, ketorolac, or ondansetron in a retrospective analysis of 1680 abdominal appendectomy patients. Opioid dosage by weight was likewise not significantly different for White and Black children [21]. Similarly, researchers found no racial or ethnic disparity in the administration of intraoperative morphine in a retrospective assessment of 21,000 procedures conducted at a single institution [22]. There was a significant under-representation of Black children and an over-representation of Asian and Hispanic children in the study sample, making it difficult to generalize the study findings to other institutions. However, in comparison to White children, children who were classed as Asian, Hispanic, or Pacific Islander were much less likely to be given non-opioid analgesics [9].

Research on adults has revealed racial and ethnic differences in the use of general anesthesia [23]. Unfortunately, there has been a shortage of research involving children. Among children younger than 19 years old, a recent retrospective analysis involving approximately 34,000 patients at one institution found no racial or ethnic differential in the use of intraoperative regional anaesthesia, whether it be peripheral nerve block or neuraxial block [24]. The study failed to consider critical microsystem variables associated with perioperative pain management that could influence the racial/ethnic breakdown of regional anesthesia rates. Anesthesiologists’ discretion in administering regional anesthesia, which may be influenced by racial biases, standardized perioperative surgery home protocols that include blocks advocated for by surgeons, and parents’ familiarity with the procedure are all potential contributors. As an example, compared to White patients, Black individuals are more prone to decline regional anaesthesia [25]. Few studies have examined the role of race in pediatric patients’ decisions to undergo regional anaesthesia or not. A retrospective analysis was conducted at one institution on children younger than six years old who were provided a caudal block during urologic surgery. It was discovered that parents of White children were more likely to consent to caudal block than parents of Black and Latinx children [26]. Regarding parental consent, the authors did not see any difference based on primary language. It is hard to explain the process underlying this gap because, like prior studies, this one did not elucidate the microsystem components (anesthesiologist communication, family understanding, etc.). Hospitals often treat patients from racial and ethnic backgrounds that do not reflect the country, which limits the generalizability of much research that only investigate one institution.

### 4.2 Postoperative Racial Disparities

Even though anaesthesiologists rarely see their patients again after surgery, it is critical for them to comprehend how a patient’s race affects the trajectory of their procedure. Recently, researchers looked at data from 250,000 pediatric general surgery patients in the Kids’ Inpatient Database across the country and found that racial differences in surgical morbidity were statistically significant. Specifically, the rate of complications was highest among Black patients (6.1%), followed by Hispanics (4.4%), and Whites (4.3%) [27]. As part of the Healthcare Cost and Utilization Project (HCUP), states that participate in the Kids’ Inpatient Database (KID) provide a sample of pediatric discharges. This trend of minority children having worse surgical outcomes has been confirmed in other fields as well. Postoperative pulmonary problems [28,29], cardiovascular complications and cardiac arrest [28, 29], surgical wound complications [29,30], unplanned reoperation [30], and readmission is more common in children from minority groups. Disparities in postoperative death rates are much more worrisome. There is evidence that both healthy and unwell Black children have a higher postoperative death rate than White children [28,30]. It is still not well known what causes these differences in postsurgical mortality.

Many studies have shown that minority children have a longer duration of stay following surgery, which is related to disparities in postoperative problems [27, 28]. Infants from under-represented groups may also require more time in the hospital prior to surgery. For instance, when comparing White and Black newborns with pyloric stenosis, the length of stay before and after surgery is much longer for the former [31]. Hospital stays were 13% longer for Black patients compared to White patients, 7% longer for Hispanic children, and 7% longer for Asian children, according to big national database research of about 250,000 pediatric general surgery patients in the Kids’ Inpatient Database [27]. Minority health care expenses are disproportionately higher, as expected. Hispanic children incurred 33% more hospital expenditures than White children, Asian children 20% more, and Black children 16% more, according to the same study of pediatric general surgery patients [27]. Another study that made use of the Kids’ Inpatient Database examined over 9,000 patients who had orthognathic surgery. Not only did they discover that hospital length of stay was substantially longer for Black and Hispanic patients, but hospital expenses were significantly higher for all racial groups as well [32]. Hospital expenditures for minority children are likely to be higher even when the length of stay is not lengthened. While children of different races had comparable lengths of stay in the hospital, Hispanic children had much higher expenses compared to White children, according to an analysis of the KID for 2012 that included 3400 adolescents with idiopathic scoliosis who underwent posterior spinal fusion [33]. Just recently, another study found that racial differences in appendectomy-related complications cost over $60,000,000 from 2001 to 2018 [34].

### 4.3 Future Perspectives

Instead of being issues with individuals, health disparities are more often caused by systemic injustices. This means that a system-based approach is necessary to address the issue of pediatric perioperative health disparities. Health and wealth disparities will persist in every society that is structured according to a specific social hierarchy. A crucial initial stage is to recognize that “no one individual is at fault.” Nevertheless, perioperative care takers should not passively accept this defective system. We are all directly or indirectly responsible for breaking the racist loop that permeates healthcare. It is imperative that anaesthesiologists, as perioperative doctors, take a more active role in addressing racial inequalities in pediatric perioperative care. Healthcare disparities are real, and we need to be ready to have the tough talk about them. We also need to examine how our own prejudices interact with the ways in which our profession contributes to these problems. These discussions shouldn’t boil down to empty gestures of empathy while ignoring the facts. We should all do what we can to combat health disparities, which constitute a serious public health issue in the United States.

A possible strategy to reduce racial disparities in perioperative outcomes is to apply routes such as Enhanced Recovery After Surgery (ERAS) and Perioperative Surgical Home (PSH). The ERAS and PSH protocols are founded on evidence and include standardised goals for postoperative recovery, drug management during surgery, and preoperative preparation respectively [35]. Patients should be able to return to their preoperative functional level as soon as possible and without incident according to these structured regimens. Thus, ERAS and PSH can reduce healthcare expenditures, morbidity, and hospital stays. Studies have demonstrated that standardized care regimens can help bring down surgical outcome discrepancies, such as pain and recovery time [35, 36].

By preventing healthcare practitioners from placing too much emphasis on patient characteristics like race, ethnicity, or primary language, standardised treatment regimens show promise as instruments for reducing racial disparities. An individual’s “attitudes and stereotypes that impact understanding, actions, and decisions unconsciously” are known as implicit biases [37]. Implicit biases affect everyone, including healthcare providers. Most healthcare providers, for example, favor White patients over Black patients [38]. Care regimen standardization is important for guaranteeing equity in treatment since implicit biases are more likely to impact perceptions and treatment decisions when people are mentally exhausted and stressed. A tool like the Implicit Association Test can help anesthesiologists become more self-aware of their biases [39]. When people are self-aware of their biases, they can learn to control their emotions, shift their focus to other people’s perspectives, and form partnerships whenever their biases surface [40]. These cognitive skills, when instilled in employees at the organizational level, can shield minorities from adverse effects caused by bias in healthcare [41].

## 5. Association between ethnicity and the provision of care in obstetric anesthesia

In the United Kingdom, women of various ethnic backgrounds have varied rates of adverse pregnancy and neonatal outcomes. Stillbirth, premature labor, and fetal development restriction are more common in South Asian and Black women than in white women, and the maternal mortality rate is four times greater in Black women than in white women [42]. A UK survey found that compared to white women, women from ethnic minorities had a worse experience with maternity care [43]. Nevertheless, no research has examined the correlation between ethnicity and general obstetric anesthetic treatment in the United Kingdom. American studies have shown that compared to white women, Black American women are more likely to undergo general anesthesia for caesarean sections and less likely to have neuraxial labor analgesia [23, 44]. In the United States, healthcare access and quality may be affected by an individual’s financial situation. This is in stark contrast to the United Kingdom, where all women who are eligible for NHS care receive free and universal maternity care at the point of service.

Women of different ethnic groups had different rates of general anesthesia for caesarean births and neuraxial anesthesia for vaginal births, according to our study of English births that adjusted for possible confounders and covered the years 2011–2021. The disparity in the use of neuraxial anesthesia among ethnic groups could not be adequately explained by the variation in the prevalence of aided vaginal births.

An increased risk of unfavorable maternal outcomes was linked to needless general anesthetics, according to a prior analysis of 466,014 women who had cesarean deliveries [3]. Depending on the woman’s preferences, the World Health Organization recommends labor epidural analgesia for healthy women [45]. The use of labor epidural analgesia was linked to a slight reduction in unfavorable childhood developmental outcomes, according to recent large population cohort research [46]. Therefore, one ethnic group may be at a disadvantage due to the lower rates of spinal or epidural use (especially for unassisted vaginal births) and the higher rates of general anesthesia for caesarean births (especially for planned cesarean births). A gap exists when there is a negative discrepancy in the health condition or results of different populations. A gap is considered ‘meaningful’ according to the US Agency for Healthcare Research and Quality when the relative difference between two groups is greater than 10% [47]. Although there may be personal reasons for a preference for general anesthesia during a caesarean section or for not receiving labor epidural analgesia, it is puzzling that these reasons should show up as disparities in the rates of anesthesia provision across ethnic groups.

Compared to white British women, women from the Caribbean or Black British descent were 10% more likely to have general anesthesia for an emergency caesarean section and 58% more likely to have it for an elective cesarean section, according to a single study [48]. The greater rate of general anesthesia in emergency caesarean births was only noticed after adjusting for factors including deprivation and region of residence. The correlation between socioeconomic position and access to prenatal care and the frequency and severity of cesarean sections may explain this. German cohort research group found that the likelihood of an emergency caesarean section was higher in households with lower incomes [48]. Researchers in France found that women from lower socioeconomic backgrounds were less likely to attend prenatal education, and that the likelihood of a caesarean section was higher among women who did not take part in such programs [49].

When compared to white British women, Asian or Asian British women of Pakistani or Bangladeshi descent who gave birth vaginally (not including those who had assisted vaginal deliveries) were 15% less likely to get neuraxial anesthesia, and 24% less likely, respectively.

Most research on the topic of ethnicity and obstetric anesthetic care has originated in the United States. An American study found that compared to white American women, Black American women had an adjusted odds ratio of 1.7 (1.5–1.8) for general anesthesia in caesarean births, out of 50,974 women who underwent the procedure [5]. Black women were more likely to have general anesthesia for both planned and unplanned caesarean sections than white women in the United Kingdom, according to our study of women who gave birth by this method.

Among 81,883 women whose medical records were reviewed in a perinatal database in New York State from 1998 to 2003, another American study found that compared to non-Hispanic white women, Black American women were less likely to receive neuraxial labor analgesia (adjusted odd ratio of 0.85 [95%CI 0.78–0.93]) [21]. Black women’s lower rates of neuraxial anesthesia are partially explained by their lower rates of assisted vaginal birth, according to our study. However, this cannot be drawn from the experiences of Asian or Asian British women from Bangladesh and Pakistan, where the rates of neuraxial anesthesia were still significantly lower than those of British (white) women, even after excluding women who did not have assisted vaginal births. On the other hand, neuraxial anesthesia was more common among Irish (white) women. It is interesting to mention that a multinational survey indicated that 64.2% of nulliparous Irish women and 19.4% of nulliparous English women got epidural anesthesia. [50].

There are several obstacles that make it difficult to use previously published data from the US in UK practice. The big US studies employed data that is at least 20 years old, which might not be up to date with how things are done now. In terms of ethnic composition, the United States is vastly different from the United Kingdom. While in the United Kingdom 86% of the people are white, 3.3% are black, and 7.5% are Asian, in the United States 76.5% are white (with 57.8% being non-Hispanic white) and 12.1% are black [51]. Cultural practices and biases based on ethnicity could also vary from one country to another. In the United States, 8% of the population lacks health insurance, even though the healthcare system is privately run and accessible through private or public insurance [52]. For labor epidural analgesia, the average cost in the US is $1646 (equivalent to about £1343, €1506) [52]. Although the rates of caesarean sections in the United States (31.9%) and England (28.4%), neuraxial labor analgesia is administered at a rate of 73% in the United States and 21% in the United Kingdom, respectively [53].

To address the causes that lead to racial differences in obstetric anesthesia, one article suggested an organized approach [4]. In this context, “patient level,” “provider level,” or “system level” might designate the relevant components. Factors at the patient level include differences in understanding and access to information. When compared to white women, Black and other minority ethnic women in the United Kingdom were more likely to say they were not involved in decisions regarding their care during labor and delivery and that they did not have enough information to make choices and decisions about their care [54]. Health care providers’ implicit biases towards certain ethnic groups in their assessments of labor pain severity are one example of a provider-level factor [55]. The availability of resources, such as translation services and 24-hour access to neuraxial labor analgesia, as well as the place of birth itself are examples of system-level issues.

There is no clear explanation for the disparity in the rates of aided vaginal birth between white and black women, given the rates among Black women at hospitals in Africa that are well-resourced are comparable to those among White women in the United Kingdom [56]. According to the Millennium Cohort Study, which looked at 18,239 births in the UK between 2000 and 2002, Black women had the greatest rates of emergency caesarean sections and the lowest rates of assisted vaginal births compared to other ethnic groups [57]. This disparity may be attributable to variations in the places where Black women gave birth; for instance, if aided vaginal birth was less common in midwifery-led settings, then Black women may have given birth more frequently in those places [58]. Black women reported a 20% lower likelihood of having a choice of birth location in a 2007 UK national survey [59].

## 6. Conclusions

Based on the available research, it appears that there are racial and ethnic differences in the rates of general anesthesia provided. It is difficult to find research that focuses specifically on anesthetic disparities, particularly in the field of pediatrics. Most of the research on perioperative discrepancies concentrates on the outcomes of surgical procedures and postoperative care. It is imperative that healthcare companies, anesthesia associations, and anesthesiology departments make health equity a top priority. Therefore, it is of the utmost importance that discrepancies in the utilization of neuraxial labor analgesia be further researched and addressed. This is because neuraxial labor analgesia has the potential to serve as a safety mechanism that prevents the utilization of general anesthesia.

Considering this observation, we hypothesize that the underlying cause may be multifaceted and may entail factors that are relevant to both the patient and the therapist. There is a need for additional research to be conducted to further clarify the reasons behind the disparity in the delivery of general anesthesia and neuraxial analgesia, as well as the methods that can prevent it from occurring. Patient-centered, timely administration of neuraxial labor analgesia should be the primary focus of attention, as should the identification of actionable items among patients who do not have epidural labor analgesia on the list of priorities.

## Figures and Tables

**Figure 1:**
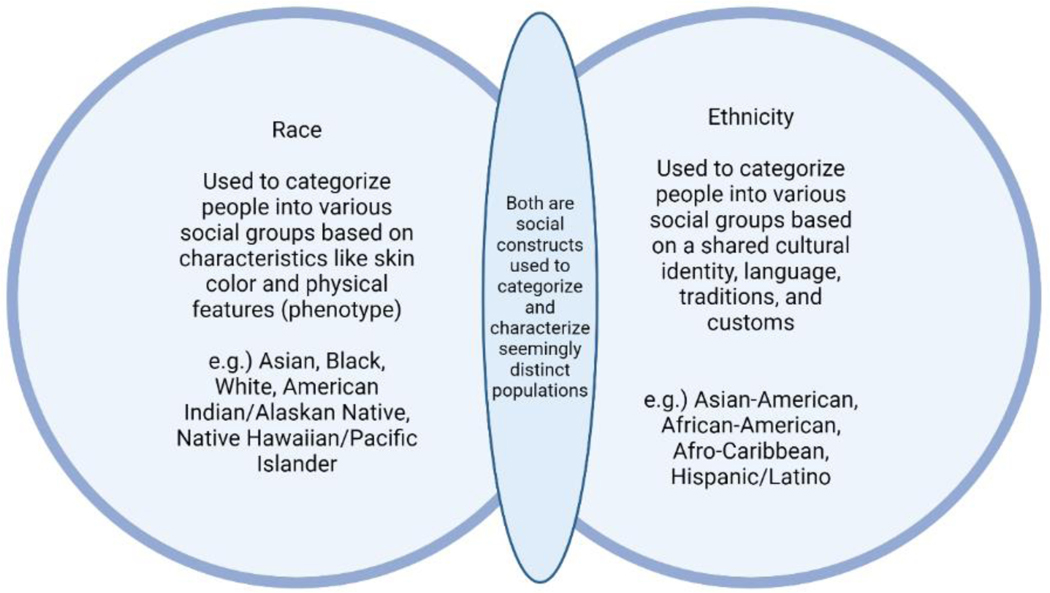
Understanding Racism in Anesthesia Care

## Key Points

- Racial and ethnic disparities affect the administration of general anesthesia for cesarean deliveries and labor analgesia.
- Black and Hispanic patients are more likely to receive general anesthesia compared to White patients, which may be linked to access to neuraxial analgesia.
- Systemic racism, implicit bias, and healthcare inequities contribute to disparities in perioperative and postoperative outcomes.
- Preoperative anxiety management and parental presence during pediatric anesthesia show discrepancies based on race, with Black children less likely to receive anxiolytics.
- Intraoperative care shows less variability in anesthesia administration across races, but post-surgical complications are higher in minority children.
- Postoperative outcomes reveal that minority children experience longer hospital stays, higher complication rates, and increased healthcare costs.
- Healthcare providers must address their own implicit biases and the systemic issues that affect the perioperative care of minority patients.
- The ERAS (Enhanced Recovery After Surgery) and PSH (Perioperative Surgical Home) protocols are suggested to reduce racial disparities by standardizing care
